# The Association Between Mild Cognitive Impairment and Medication Non-adherence Among Elderly Patients With Chronic Diseases

**DOI:** 10.7759/cureus.47756

**Published:** 2023-10-26

**Authors:** Xiaoqin He, Xinguo Wang, Bin Wang, Aiyong Zhu

**Affiliations:** 1 School of Graduate, Shanghai University of Traditional Chinese Medicine, Shanghai, CHN; 2 School of Nursing and Health Management, Shanghai University of Medicine and Health Sciences, Shanghai, CHN; 3 Department of Endocrinology and Metabolism, Affiliated Hospital of Qingdao University, Qingdao, CHN

**Keywords:** influential factor, chronic diseases, elderly, medication adherence, mild cognitive impairment

## Abstract

Background: Medication adherence is essential for optimizing treatment outcomes in elderly patients who frequently contend with multiple chronic diseases requiring pharmacological interventions. Mild cognitive impairment (MCI) is a prevalent cognitive disorder among the elderly population, but its impact on medication adherence among elderly patients is still uncertain. This cross-sectional study aimed to investigate the impact of MCI on medication adherence among elderly patients.

Methods: A cross-sectional study of 436 elderly patients with common chronic diseases aged 60 years and above was conducted. Medication adherence was measured using the Morisky Medication Adherence Scale-8 (MMAS-8). MCI was screened, and cognitive status was assessed using the Mini-Mental State Examination (MMSE) questionnaire. Multivariate logistic regression analysis was performed to identify independent risk factors of medication non-adherence.

Results: Among these elderly patients, 212 (48.6%) had poor medication compliance, and 181 (41.5%) had MCI. Preliminary analyses showed a significant association between MCI and medication non-adherence among elderly patients (odds ratio (OR)=3.95, 95% confidence interval (95%CI)=2.63-5.92, P<0.001). Multivariate logistic regression analysis showed that MCI was independently associated with the risk of medication non-adherence among elderly patients (adjusted OR=2.64, 95%CI=1.64-4.24, P<0.001). Additionally, adverse drug reaction and poor evaluation of medication effects were also independently associated with medication non-adherence in elderly patients (P<0.05).

Conclusion: Findings from this cross-sectional study proved the substantial adverse impact of MCI on medication adherence among elderly patients, and MCI was an independently influential factor of medication non-adherence. Identifying the MCI status early and providing interventions to enhance medication adherence are undoubtedly essential for optimizing healthcare outcomes in elderly patients.

## Introduction

There has been a steady increase in the elderly population worldwide during the past three decades, and it is the same with the prevalence of many chronic diseases such as diabetes and hypertension [1,2]. This significant transformation is accompanied by a rising necessity for pharmacological interventions [3]. However, the treatment outcomes of pharmacological interventions differ obviously among different individuals, and the efficacy can be influenced by some factors such as genetic or behavioral factors. Among those factors, medication adherence, defined as the extent to which patients take prescribed medications according to clinical recommendations, has long been identified to be essential for optimizing treatment outcomes [4,5]. Among elderly individuals with multiple chronic diseases who frequently require complex treatments, medication adherence plays a pivotal role in achieving optimal treatment targets [6,7]. On the contrary, medication non-adherence can result in reduced efficacy of pharmacological interventions or even treatment failure [8].

To improve medication adherence, it is crucial to identify the factors impairing adherence to pharmacological interventions and further provide additional interventions with the aim of enhancing medication adherence. Some studies have shown that medication adherence can be influenced by many factors such as disease knowledge, medication side effects, medical cost, and patient's economic status [9,10]. Those findings are helpful in identifying those individuals at high risk of medication non-adherence and the development of effective interventions for enhancing adherence to therapy [11,12]. However, many factors involved in the development of medication non-adherence are still not defined, and further research is needed.

Mild cognitive impairment (MCI) is a prevalent cognitive disorder among the elderly population, and it represents a transitional stage between normal cognitive aging and more severe forms of cognitive decline such as Alzheimer's disease [13,14]. MCI is characterized by cognitive deficits of exceeding age-related changes but not significantly impairing daily functioning. Despite the substantial attention paid to either medication non-adherence or MCI in the elderly, the interplay between these two factors is still largely elusive. There is still no conclusive answer to the questions about the potential impact of MCI on medication adherence in elderly patients. To provide insights into the impact of MCI on medication adherence among elderly patients, we conducted a cross-sectional study. In this study, the association between MCI and medication adherence was assessed. Multivariate logistic regression analysis was also performed to identify whether MCI was an independent risk factor of medication non-adherence among elderly patients.

## Materials and methods

Setting and participants

This cross-sectional study was carried out in the primary care clinics of Shanghai City between January 2022 and December 2022. The study recruited a total of 436 elderly patients aged 60 years and above. Inclusion criteria encompassed participants who were aged 60 years and above and were receiving prescribed medications for chronic conditions such as diabetes, hypertension, cerebrovascular disease, ischemic heart disease, and chronic obstructive pulmonary disease. Participants with severe dementia or individuals who were unable to provide informed consent were excluded from the study. The study received ethical approval from the Ethics Committee of the primary care institutions. Informed consent was obtained from all participants.

Assessment of cognitive status

The cognitive status of participants was assessed using the Mini-Mental State Examination (MMSE), which was a widely used and standardized cognitive assessment tool. MMSE is a cognitive assessment tool developed by Folstein et al. [15]. This tool had six domains and 30 questions and had a total score ranging from 0 to 30 points. The cutoff value for MCI depends on the education level of study participants. In this study, the cutoff value for MCI was 17 points for individuals with illiteracy, 20 points for individuals with primary school education, and 24 points for individuals with junior high school education or higher, which were consistent with the cutoff values adopted in most studies of screening cognitive status from Chinese population [16,17]. MCI was defined as an MMSE score not exceeding 17 for illiterate patients, not exceeding 20 for patients with primary education, and not exceeding 24 for patients with junior high school education or higher.

Assessment of medication adherence

Self-reported medication adherence among participants was measured using the eight-item Morisky Medication Adherence Scale (MMAS-8), which is a widely used standardized medication assessment tool [18,19]. MMAS-8 comprises a total of eight items, and all items are categorized as either "yes" or "no" with the exception of the final item that employs a 5-point rating scale. The maximum score on the scale is 8 points, with higher scores indicating better adherence. Scores below 6 points reflect poor adherence, scores ranging from 6 to 8 points indicate moderate adherence, and a perfect score of 8 points suggests good adherence. The MMAS-8 has been extensively used in numerous research studies to evaluate medication adherence among elderly individuals with chronic conditions, and the assessment results demonstrate high reliability [20,21].

Other variables and clinical data

Participants also provided information on demographic factors and clinical data such as age, gender, smoking status, education level, healthcare type, living area, medication beliefs, exercise, disease knowledge, adverse drug reaction, and tolerance of medical expenses. Most of those factors had been reported to be associated with medication adherence in some published literature. Those data were collected by structured interviews with study questionnaires for demographic and clinical characteristics.

Statistical analyses

Quantitative variables were expressed as mean ± standard deviation (SD), and discrete variables were expressed as frequency with percentages. Demographic factors, comorbidities, and other factors were also analyzed for their influence on medication adherence. Univariate logistic regression analysis was conducted, and odds ratio (OR) with 95% confidence interval (95%CI) was calculated. Multivariate logistic regression analysis was then performed to identify independent risk factors of medication non-adherence among elderly patients with chronic diseases by controlling for relevant covariates including demographic variables and comorbidities. Statistical analyses were performed using Stata software (version 12.1) (StataCorp, College Station, TX), and P<0.05 indicates statistical significance.

## Results

Participant characteristics

A total of 436 elderly patients with chronic diseases were included, and the mean age of the participants was 72.5 years, with 228 (52.3%) female participants. Table 1 shows the demographic and clinical characteristics of those 436 elderly patients with chronic diseases. Among those elderly patients, 355 (81.4%) had hypertension, 155 (35.5%) had diabetes, 136 (31.2%) had ischemic heart disease, 85 (19.5%) had cerebrovascular diseases, and 22 (5%) had chronic obstructive pulmonary disease.

**Table 1:** Demographic and clinical characteristics of the 436 elderly patients with chronic diseases

Variable	Number	Percentage (%)
Age group, number (%)		
60-69 years	169	38.8%
70-79 years	193	44.3%
≥80 years	74	16.9%
Gender, number (%)		
Male	208	47.7%
Female	228	52.3%
Smoking, number (%)		
Yes	63	14.4%
No	331	75.9%
Ever	42	9.6%
Drinking, number (%)		
Yes	30	6.9%
No	381	87.4%
Ever	25	5.7%
Living area, number (%)		
City	190	43.6%
Towns	121	27.7%
Villages	125	28.7%
Education level, number (%)		
Illiteracy	55	12.6%
Primary school	117	26.8%
Junior high school	134	30.7%
Senior high school	88	20.2%
College graduate	42	9.6%
Retirement, number (%)		
Yes	359	82.3%
No	77	17.7%
Diseases, number (%)		
Diabetes	155	35.5%
Hypertension	355	81.4%
Cerebrovascular disease	85	19.5%
Ischemic heart disease	136	31.2
Chronic obstructive pulmonary disease	22	5%
Disease duration, number (%)		
＜5 years	88	20.2%
5-9 years	86	19.7%
10-15 years	99	22.7%
16-20 years	83	19%
＞20 years	80	18.3%
Source of medical expenses, number (%)		
Medical insurance or public medical care	426	97.7%
Own expense	10	2.3%

Those 436 elderly patients included 220 (50.5%) elderly patients with at least two chronic diseases and 79 (18.1%) elderly patients with at least three chronic diseases. The average number of diagnosed chronic medical conditions per participant was 1.73, indicating that elderly patients suffering from multimorbidity were very common, and the degree of complexity of medication was high.

Based on the cognitive assessment score by MMSE and the established criteria for MCI, 255 cases had normal cognitive function, accounting for 58.5% of the total, while 181 cases exhibited MCI, representing 41.5% of the total.

Medication adherence

The mean total MMAS-8 score for medication adherence among these 436 elderly patients with chronic diseases was 5.90 points (SD=1.61) (Table 2). The mean scores for each item are shown in Table 2. Among the 436 elderly patients with chronic diseases, 212 had poor medication adherence (score below 6 points), accounting for 48.6% of the total. A total of 141 (32.3%) elderly patients had poor medication adherence (scores ranging from 6 to 8 points), but only 83 (19%) had good medication adherence (score of 8 points). Therefore, the proportion of medication non-adherence in these 436 elderly patients with chronic diseases was relatively high.

**Table 2:** Assessment outcomes of medication adherence by MMAS-8 among the 436 elderly patients with chronic diseases MMAS-8: Morisky Medication Adherence Scale-8, SD: standard deviation

Item	Mean±SD
1. Do you sometimes forget to take your hypertension medications?	0.40±0.49
2. Thinking over the past two weeks, were there any days when you did not take your medications?	0.70±0.46
3. Have you ever cut back or stopped taking your medication without telling your doctor because you felt worse when you took it?	0.80±0.40
4. When you travel or leave home, do you sometimes forget to bring along your medications?	0.88±0.32
5. Did you take your medications yesterday?	0.96±0.20
6. When you feel like your disease is under control, do you sometimes stop taking your medications?	0.81±0.39
7. Do you ever feel hassled about sticking to your treatment regimen?	0.57±0.50
8. How often do you have difficulty remembering to take all your medications?	0.77±0.24
Total score	5.90±1.61

Risk factors associated with medication adherence

Preliminary analyses via univariate logistic regression showed a significant association between MCI and medication non-adherence among elderly patients (OR=3.95, 95%CI=2.63-5.92, P<0.001) (Table 3). A total of 14 other factors, such as disease duration, lack of regular exercise, multimorbidity, poor disease knowledge, adverse drug reaction, and tolerance of medical expenses, were also found to be associated with medication non-adherence (P<0.05) (Table 3).

**Table 3:** Outcomes in the univariate logistic regression analysis to identify factors associated with medication adherence in elderly patients with chronic diseases OR: odds ratio, 95%CI: 95% confidence interval, SE: standard error, MCI: mild cognitive impairment

Variable	OR	95%CI	SE	z	P
Age (high versus low)	1.22	0.94-1.59	0.16	1.50	0.134
Gender (female versus male)	0.93	0.64-1.36	0.18	-0.36	0.721
Smoking (yes versus no and ever)	0.82	0.48-1.40	0.22	-0.72	0.473
Drinking (yes versus no and ever)	0.79	0.37-1.68	0.30	-0.60	0.549
Disease duration (high versus low)	1.16	1.01-1.33	0.08	2.15	0.032
Living area	0.80	0.64-1.01	0.09	-1.87	0.061
Education level (high versus low)	1.10	0.94-1.30	0.09	1.22	0.224
Retirement (no versus yes)	1.03	0.63-1.69	0.26	0.14	0.888
Medical insurance (no versus yes)	4.35	0.91-20.73	3.46	1.85	0.065
Regular exercise (no versus yes)	1.56	1.05-2.31	0.31	2.22	0.026
Recreational activities (no versus yes)	1.64	1.12-2.42	0.32	2.53	0.011
Multimorbidity (yes versus no)	2.36	1.60-3.46	0.46	4.38	<0.001
Disease knowledge (good versus poor)	0.52	0.39-0.71	0.08	-4.18	<0.001
Hearing loss (yes versus no)	3.17	1.77-5.67	0.94	3.89	<0.001
Types of medications per day	1.52	1.29-1.79	0.12	5.11	<0.001
Frequency of medications per day	1.75	1.45-2.13	0.17	5.77	<0.001
Adverse drug reaction (yes versus no)	4.58	2.99-7.01	0.99	7.01	<0.001
Self-medication (no versus yes)	2.61	1.57-4.33	0.67	3.72	<0.001
Education of medication knowledge (yes versus no)	0.68	0.51-0.91	0.10	-2.56	0.010
Frequency of drug purchase (low versus high)	0.66	0.51-0.86	0.09	-3.07	0.002
Tolerance of medical expenses (low versus high)	1.88	1.46-2.41	0.23	4.99	<0.001
Evaluation of medication effect (poor versus good)	2.18	1.68-2.81	0.28	5.98	<0.001
MCI (yes versus no)	3.95	2.63-5.92	0.81	6.66	<0.001

A total of 15 factors including MCI with a P value of less than 0.05 were used in the multivariate logistic regression analysis, while the remaining factors were omitted. After adjusting for potential confounders, outcomes from multivariate logistic regression analysis showed that MCI was independently associated with the risk of medication non-adherence among elderly patients with chronic diseases (OR=2.64, 95%CI=1.64-4.24, P<0.001) (Table 4). This suggested that elderly patients with MCI were 2.64-fold more likely to exhibit medication non-adherence compared to their cognitively intact controls. Additionally, adverse drug reaction and poor evaluation of medication effects were also independently associated with medication non-adherence in elderly patients with chronic diseases (P<0.05) (Table 4).

**Table 4:** Outcomes in the multivariate logistic regression analysis to identify independently influential factors of medication adherence in elderly patients with chronic diseases OR: odds ratio, 95%CI: 95% confidence interval, SE: standard error, MCI: mild cognitive impairment

Variable	OR	95%CI	SE	z	P
Disease duration (high versus low)	0.96	0.80-1.15	0.09	-0.46	0.647
Regular exercise (no versus yes)	0.98	0.59-1.63	0.26	-0.08	0.935
Recreational activities (no versus yes)	0.93	0.57-1.54	0.24	-0.27	0.788
Multimorbidity (yes versus no)	1.07	0.58-1.96	0.33	0.22	0.823
Disease knowledge (good versus poor)	0.77	0.53-1.13	0.15	-1.33	0.183
Hearing loss (yes versus no)	1.92	0.96-3.85	0.68	1.84	0.065
Types of medications per day	1.00	0.72-1.39	0.17	-0.02	0.986
Frequency of medications per day	1.26	0.90-1.76	0.22	1.33	0.185
Adverse drug reaction (yes versus no)	2.25	1.35-3.74	0.58	3.13	0.002
Self-medication (no versus yes)	1.17	0.69-1.98	0.31	0.59	0.554
Education of medication knowledge (yes versus no)	0.73	0.51-1.03	0.13	-1.79	0.074
Frequency of drug purchase (low versus high)	0.81	0.59-1.12	0.13	-1.28	0.202
Tolerance of medical expenses (low versus high)	1.21	0.88-1.67	0.20	1.17	0.242
Evaluation of medication effect (poor versus good)	1.58	1.17-2.13	0.24	3.01	0.003
MCI (yes versus no)	2.64	1.64-4.24	0.64	4.01	<0.001

## Discussion

Medication adherence is essential for optimizing treatment outcomes in elderly patients with chronic diseases requiring pharmacological interventions. Meanwhile, MCI is a prevalent cognitive disorder among the elderly population, but its impact on medication adherence among elderly patients is still uncertain. Cognitive deficits associated with MCI, such as memory impairment and executive dysfunction, may have the potential to hinder the ability of these elderly patients to use their medication regimens adequately. In this study, we carried out a cross-sectional study to evaluate the impact of MCI on the medication adherence of elderly patients. Findings from this cross-sectional study proved the substantial adverse impact of MCI on medication adherence among elderly patients, and MCI was an independently influential factor of medication non-adherence among elderly patients with chronic diseases.

The findings from this study underscore the significant impact of MCI on medication adherence among elderly patients, shedding light on the challenges faced by elderly individuals with cognitive impairment. Forgetfulness, misinterpretation of drug instructions, and difficulty in coordinating complex dosing schedules in MCI elderly patients can all contribute to suboptimal medication adherence [20-23]. Identifying the MCI status early and providing early interventions are essential for optimizing healthcare outcomes in elderly patients with chronic diseases [24-26].

Identifying MCI early in its course allows for tailored interventions, such as simplified medication regimens, medication reminders, and caregiver involvement, which may enhance adherence and subsequently improve healthcare outcomes [27,28]. Clinicians should be vigilant in assessing and addressing cognitive impairment in these elderly patients with chronic diseases to improve medication management and ensure treatment outcomes.

The study also revealed the influence of other factors on medication adherence such as adverse drug reaction and poor evaluation of medication effects. These findings emphasize the importance of considering not only cognitive status but also other factors when designing interventions to promote medication adherence in the elderly [29,30].

There were some limitations in our study. As our study used a cross-sectional design, it had limitations in determining causal relationships between MCI and non-adherence. Moreover, there was a possibility of selection bias and recall bias, which could result in reduced generalizability in our study. The impact of MCI on non-adherence still needs to be evaluated by prospective cohort studies with long-term follow-up and a larger number of participants.

## Conclusions

Findings from this cross-sectional study proved the substantial adverse impact of MCI on medication adherence among elderly patients, and MCI was an independently influential factor of medication non-adherence. Identifying the MCI status early and providing interventions to enhance medication adherence are undoubtedly essential for optimizing healthcare outcomes in elderly patients with chronic diseases. Future research focusing on developing effective interventions for improving medication adherence in elderly individuals with MCI is warranted.
